# A Comprehensive Approach to Enhance Older Adults’ Preparedness for Extreme Heat: COPE -Engage

**DOI:** 10.1093/geroni/igaf122.1430

**Published:** 2025-12-31

**Authors:** Atiya Mahmood, Leticia Zhu, Cindy Wei, Theresa Pauly

**Affiliations:** Simon Fraser University, Vancouver, British Columbia, Canada; Simon Fraser University, Vancouver, British Columbia, Canada; Simon Fraser University, Vancouver, British Columbia, Canada; Simon Fraser University, Vancouver, British Columbia, Canada

## Abstract

Climate change has resulted in increase in the frequency of extreme heat days and older adults are disproportionately affected due to physiological vulnerabilities and systemic barriers to adaptation. In this study, we conduct focus group interviews with older adults and semi-structured interviews with community partners (e.g., healthcare and housing providers, city planners and community service organizations) to identify best practices and challenges in existing heat response strategies. Focus groups findings provide rich, narrative-driven insights into lived experiences, enabling us to understand how older adults interpret and respond to heat-related risks as they navigate extreme heat and access community services. Questions are asked about awareness and preparedness; perception of vulnerability; socioeconomic influence; thermal comfort and heat relief. Findings from the semi-structured interviews provide in-depth information on how public organizations and service sectors deliver service and programs during these events. The community partners are asked about access and quality of community facilities, services and programs to mitigate and manage extreme heat events. Preliminary findings demonstrate that older adults who are supported through specific community-based organizations during heat events have better awareness about risk factors and are able to access and use services more effectively. Transportations play a key role in utilization of services like cooling centres. Community organizations emphasize the need for more multi-sectorial collaboration to better serve older adults. Both groups highlight the need for better communication and awareness campaigns around risks factors of extreme heat event. A multi-year approach integrates co-creation workshops to refine intervention strategies based on study findings.

